# Supplementary material for: A hierarchy of timescales explains distinct effects of local inhibition of primary visual cortex and frontal eye fields
Source: eLife. 2016 Sep 6;5:e15252. doi: 10.7554/eLife.15252 (PMC5012863; doi:10.7554/eLife.15252)
Supplement: Supplementary file 1. — DOI: http://dx.doi.org/10.7554/eLife.15252.019 [file elife-15252-supp1.docx]

**Supplementary file 1: Changes in TMS-targets to whole brain functional connectivity**

|  | **Anatomy ^a^** | | | **Stats ^b^** | | | | |
| --- | --- | --- | --- | --- | --- | --- | --- | --- |
|  | **x** | **y** | **z** | **K_E_** | **Z** | | **P_corr_** | |
| ***Regions showing increased positive correlations with the right V1/V2 after stimulation*** | | | | | | | | |
| Superior and middle frontal gyri | 30 | 6 | 45 | 668 | | 3.99 | | 0.05  (0.02 FDR) |
|  | 18 | 18 | 45 |  |  | 3.19 | |  |
|  | -12 | 0 | 69 |  |  | 2.96 | |  |
|  | -6 | 9 | 51 |  |  | 2.48 | |  |
| Lingual gyrus | 24 | -45 | -6 | 2216 | | 3.76 | | < 0.001 |
|  | -18 | -60 | 3 |  |  | 3.35 | |  |
|  | -21 | -57 | -3 |  |  | 3.24 | |  |
| Lateral occipital cortex/Cuneus | 33 | -75 | 24 |  |  | 3.03 | |  |
| Parietal cortex | 24 | -75 | 48 |  |  | 2.91 | |  |
| Middle occipital gyrus / descending occipital gyrus | 42 | -75 | -3 |  |  | 2.93 | |  |
|  | -42 | -78 | -6 |  |  | 2.42 | |  |
| ***Regions showing reduced correlations with right FEF after stimulation*** | | | | | | | | |
| Fusiform gyrus | -18 | -81 | 9 | 577 | | 3.04 | | 0.003 |
|  | -36 | -66 | -6 |  |  | 3.05 | |  |
| Occipital pole / descending occipital gyrus / inferior occipital gyrus | -24 | -96 | -9 |  |  | 2.69 | |  |
| Occipital pole | 18 | -93 | -9 | 1038 | | 3.61 | | < 0.001 |
| Cuneus / middle occipital gyrus | 24 | -84 | 6 |  |  | 3.88 | |  |
|  | 27 | -69 | 24 |  |  | 3.30 | |  |
| Inferior / middle occipital gyrus | 30 | -87 | 0 |  |  | 3.18 | |  |
| Middle / superior occipital gyrus | 30 | -87 | 18 |  |  | 2.96 | |  |

**Note**. **^a^** Coordinates (x, y, z) are given in Montreal Neurological Institute (MNI) atlas space. **^b^** *p* values are family-wise error (FWE) corrected for multiple comparisons at the cluster level (p< 0.05), unless otherwise specified. FEF = frontal eye fields.
