# Supplementary material for: A hierarchy of timescales explains distinct effects of local inhibition of primary visual cortex and frontal eye fields
Source: eLife. 2016 Sep 6;5:e15252. doi: 10.7554/eLife.15252 (PMC5012863; doi:10.7554/eLife.15252)
Supplement: Supplementary file 2. — Group-level average values extracted from key regions showing a significant change in functional connectivity after inhibitory TMS (Figure 3). Standard error of the mean (in brackets) was calculated on single-subject connectivity values in each region. Regions consisted of a sphere of 7.5mm of radius centered on the co-ordinates indicated (MNI space). DOI: http://dx.doi.org/10.7554/eLife.15252.020 [file elife-15252-supp2.docx]

**Supplementary file 2: Direction of changes between regions targeted with TMS and whole brain functional connectivity**

**V1/V2 Stimulation**

| **V1/V2 to** | **Lingual gyrus**  **(x=24, y=-45, z=-6)** | **Lingual gyrus**  **(x=-24,y=-45,z=-6)** | **Lateral occipital cortex**  **(x=33,y=-75,z=24)** | **Superior and middle frontal gyri**  **(x=30,y=6,z=45)** | **Superior and middle frontal gyri**  **(x=-12,y=0,z-=69)** |
| --- | --- | --- | --- | --- | --- |
| **Pre-TMS connectivity** | -0.007  (±0.027) | -0.021  (±0.028) | 0.026  (±0.024) | -0.066  (±0.019) | -0.005  (±0.02) |
| **Post-TMS**  **connectivity** | 0.109  (±0.030) | 0.113  (±0.033) | 0.066  (±0.024) | 0.023  (±0.018) | 0.048  (±0.022) |

**FEF Stimulation**

| **FEF to** | **Occipital pole**  **(x=-24,y=-96,z=-9)** | **Occipital pole**  **(x=18,y=-93,z=-9)** |
| --- | --- | --- |
| **Pre-TMS**  **connectivity** | 0.139  (±0.033) | 0.126  (±0.036) |
| **Post-TMS**  **connectivity** | 0.031  (±0.027) | 0.005  (±0.028) |

**Note**: Group-level mean values extracted from key regions showing a significant change in functional connectivity after inhibitory TMS (Fig. 3). Standard error of the mean (in brackets) was calculated on single-subject connectivity values in each region. Regions consisted of key clusters presented in Fig.3 (local maxima co-ordinates are indicated, MNI space).
