# Supplementary material for: A hierarchy of timescales explains distinct effects of local inhibition of primary visual cortex and frontal eye fields
Source: eLife. 2016 Sep 6;5:e15252. doi: 10.7554/eLife.15252 (PMC5012863; doi:10.7554/eLife.15252)
Supplement: Supplementary file 3. — DOI: http://dx.doi.org/10.7554/eLife.15252.021 [file elife-15252-supp3.docx]

|  | ***V1/V2*** | | |  | ***FEF*** | | |
| --- | --- | --- | --- | --- | --- | --- | --- |
| *Subject ID* | *Pre* | *Post* | *Difference* |  | *Pre* | *Post* | *Difference* |
| 1 | -0.4139 | 0.3794 | -0.7933 |  | -0.3608 | -0.3938 | 0.0330 |
| 2 | 0.2418 | -0.1421 | 0.3839 |  | 0.1007 | 0.0425 | 0.0582 |
| 3 | 1.0315 | 0.3678 | 0.6637 |  | -0.0382 | -0.2766 | 0.2384 |
| 4 | -0.3204 | -0.2887 | -0.0317 |  | -0.2227 | -0.0689 | -0.1538 |
| 5 | -0.4275 | -0.0222 | -0.4053 |  | -0.1924 | -0.1825 | -0.0099 |
| 6 | 0.5397 | 0.5349 | 0.0048 |  | -0.4084 | -0.2334 | -0.1750 |
| 7 | 0.3274 | 0.7090 | -0.3816 |  | -0.0435 | -0.2028 | 0.1593 |
| 8 | 0.3483 | 0.8689 | -0.5206 |  | -0.2194 | -0.1181 | -0.1013 |
| 9 | 0.7839 | 0.2259 | 0.5580 |  | -0.3049 | -0.2844 | -0.0205 |
| 10 | 0.3527 | 0.6979 | -0.3452 |  | -0.5885 | -0.5398 | -0.0487 |
| 11 | 0.6819 | 0.9338 | -0.2519 |  | -0.3406 | -0.6126 | 0.2720 |
| 12 | 0.5098 | 0.5726 | -0.0628 |  | -0.3334 | -0.3068 | -0.0266 |
| 13 | 1.4034 | 1.3864 | 0.0170 |  | -0.3599 | -0.4433 | 0.0834 |
| 14 | 0.7657 | 0.7380 | 0.0277 |  | -0.5086 | -0.3533 | -0.1553 |
| 15 | 1.2631 | 1.5573 | -0.2942 |  | -0.3149 | -0.5129 | 0.1980 |
| 16 | -0.3317 | 0.0403 | -0.3720 |  | -0.4880 | -0.5672 | 0.0792 |
| 17 | -0.0023 | 0.0051 | -0.0074 |  | -0.2465 | -0.3405 | 0.0940 |
| 18 | 0.4489 | 0.3849 | 0.0640 |  | 0.0085 | 0.0215 | -0.0130 |
| 19 | 0.9573 | 0.5612 | 0.3961 |  | -0.3651 | -0.3762 | 0.0111 |
| 20 | 0.3570 | 0.3699 | -0.0129 |  | -0.4202 | -0.3899 | -0.0303 |
| 21 | 0.4439 | 0.6830 | -0.2391 |  | -0.1697 | -0.1309 | -0.0388 |

**Supplementary file 3: ALFF values**
